# Differentiating Essential and Dystonic Head Tremor: Exploring Arm Position Effects

**DOI:** 10.1002/mdc3.14269

**Published:** 2024-11-15

**Authors:** Tereza Hubená, Petr Hollý, Aneta Pavlíková, Olga Ulmanová, Jan Rusz, Radim Krupička, Evžen Růžička

**Affiliations:** ^1^ Department of Biomedical Informatics Czech Technical University in Prague, Faculty of Biomedical Engineering Kladno Czech Republic; ^2^ Department of Neurology and Centre of Clinical Neuroscience Charles University, First Faculty of Medicine and General University Hospital in Prague Prague Czech Republic; ^3^ Department of Circuit Theory Czech Technical University in Prague, Faculty of Electrical Engineering Prague Czech Republic

**Keywords:** coherence, dystonic tremor, essential tremor, inertial measurement unit, tremor stability index

## Abstract

**Background:**

Head tremor poses diagnostic problems, especially when present as an isolated or predominant symptom.

**Objectives:**

To assess how maneuvers activating upper limb postural tremor can help differentiate head tremor in essential tremor (ET) from dystonic tremor (DT) in cervical dystonia.

**Methods:**

48 patients with head tremor (25 ET, 23 DT), underwent clinical examination and accelerometric evaluation of head and upper limb tremor during routine tremor‐inducing tasks.

**Results:**

While accelerometric power and clinical scores of head tremor did not significantly differ between patient groups, task‐induced variations revealed distinctions. ET patients exhibited increased head tremor power and clinical scores during forward outstretched and lateral wing‐beating arm positions, unlike DT patients. Coherence between head and upper limb tremor remained consistent. Tremor stability index showed no significant differences.

**Conclusions:**

Task‐induced changes in head tremor could aid in distinguishing between ET and DT. Further research is needed to refine diagnostic approaches for head tremor.

Essential tremor (ET) is defined as an isolated syndrome of bilateral upper limb action tremor with at least 3 years’ duration, possibly accompanied by midline tremors (head, vocal cords, and face), in the absence of abnormal posturing, task specificity, or position dependence.^1^ However, there is a significant proportion of patients with a predominant or isolated head tremor who don't fulfill the current diagnostic criteria for ET.^2^ In differential diagnosis, dystonic tremor (DT) in cervical dystonia should be considered, especially when there is a head tremor in combination with dystonic postures of the head and neck.^1^ Dystonia frequently presents with tremor,^3^ which is part of isolated dystonia.^4^ DT tends to exhibit variability across different tasks^5^ and typically worsens when the patient attempts to counteract the dystonic torsion voluntarily. Clinical differentiation of ET and DT is complicated by the fact that mild dystonia can be easily overlooked,^6^ and cervical dystonia can develop years after the onset of head tremor.^7^

Various clinical and neurophysiological tests have been suggested to support the differential diagnosis of tremor.^8^ Studies using inertial sensors calculate parameters assessing tremor frequency, amplitude and frequency regularity,^9^, ^10^, ^11^, ^12^ alongside employing Principal Component Analysis (PCA) to identify primary contributors to tremor.^11^ Tremor coherence, which tends to be lower in DT compared to ET, has been also considered.^9^ Furthermore, research indicates that tremor frequency irregularity is more pronounced in DT than in ET,^9^, ^13^, ^14^ with evidence suggesting that irregularity is independent of tremor amplitude.^13^

Attempts to better identify the origin of head tremor using clinical examination maneuvers have yielded limited success. While some studies have shown relief of dystonic head tremor but not ET with sensory tricks,^15^ the reliability of this approach has been questioned.^16^ Conversely, reports indicate that while essential head tremor disappears in the supine position, dystonic head tremor persists.^17^ Thus, a reliable diagnostic method to distinguish DT from ET of the head remains elusive.

By definition, one would expect that, unlike dystonic tremor, head tremor in ET should not respond to positional tasks.^1^ However, in clinical testing of patients with ET, we occasionally noticed that the severity of head tremor varied depending on the position of their arms. Therefore, we undertook the present study to test the differential diagnostic utility of some of the tests and maneuvers used in the clinical examination of tremor by visual assessment and accelerometric measurement of head tremor.

## Methods

### Patients

Patients with head tremor were recruited at the Movement Disorders Centre, Department of Neurology, General University Hospital in Prague. In total, 48 patients were included, 25 patients (15 women and 10 men, mean age 67.3 ± 11.9 years) with ET diagnosed according to the current classification criteria,^1^ and 23 patients (18 women and 5 men, mean age 64.5 ± 10.8 years) with cervical dystonia and dystonic head tremor.^4^ Patients with a known genetic or secondary origin of dystonia and those with known comorbidity affecting the position and movements of the neck or upper limbs (UL) were not included. One patient with ET (4%) and 22 patients with DT (96%) were receiving botulinum toxin (BTX) injections, with testing scheduled at least 12 weeks after the last dose.

### Clinical Examination

Every participant was examined by a neurologist (PH), using a structured questionnaire covering a family history of tremor, the patient's disease symptoms, their progression and comorbidities. The patient's condition was evaluated according to the Essential Tremor Rating Assessment Scale (TETRAS).^18^ The score of UL action tremor was computed by aggregating the TETRAS Performance Subscale (PS) item 4 subscores for UL tremor on both the right and left sides, encompassing forward outstretched postural tremor, lateral wing‐beating postural tremor, and kinetic tremor. Furthermore, the patients were assessed according to the Toronto Western Spasmodic Torticollis Rating Scale (TWSTRS).^19^

### Protocol

Inertial measurement units (MTw Awinda, Xsens, the Netherlands) were fixed on the patient's forehead and the dorsum of both hands using Xsens gloves. Head and UL tremor was recorded in the sitting position with relaxed hands placed on the thighs during rest (SRest), phonation (SPhon) and cognitive task (SCogn); with the upper limbs outstretched forward (SForw) and in the wing position (SWing) (Fig. S1). Recordings were at least 20 s long for each task. A spatially defined frontal view video recording of each patient was taken during the examination.

### Data Analysis

The power of the tremor was calculated using Welch's power spectral density estimation of the acceleration in the *x*, *y*, and *z* axes and combined into a single spectrum using the Euclidean norm. Coherence and its significance^20^ were calculated between head and right hand, head and left hand and between hands. Tremor frequency stability was computed using the Tremor stability index (TSI).^12^

Two blinded raters (ER and OU) independently evaluated anonymized patient videos, assessing head tremor severity for each task using TETRAS PS item 1. The average scores were used for further calculations.

Further details on data analysis and statistics can be found in the Supplementary Methods.

## Results

There were no significant differences between ET and DT patients in the age, age at onset, or family history of tremor. All patients with ET and 13 out of 23 (57%) patients with DT had UL action tremor (Table 1). Resting UL tremor was found in 12/25 (48%) patients with ET and 1/23 (4%) patients with DT. In addition, three patients in the ET group were found to have slight head deviation rated at a maximum of one point in each of the TWSTRS subscales for rotation, laterocollis, and antecollis. Consistently, ET patients exhibited higher average overall TETRAS scores and UL tremor subscores, while TWSTRS scores were higher in patients with DT (Table 1).

**TABLE 1:** Scores of the clinical scales. Results are shown as median (IQR) or as a ratio of person numbers (percentage).

	ET	DT	*P* values
Positive family history	16/25 (64%)	10/23 (43%)	0.246
Age at onset	35.0 (34.0)	41.0 (19.0)	0.374
TETRAS total score	43.5 (27.0)	11.3 (9.1)	<0.001
TETRAS ADL	24.0 (17.0)	6.0 (4.5)	<0.001
TETRAS PS	18.3 (8.5)	4.5 (7.0)	<0.001
TETRAS head tremor	2.0 (2.0)	2.0 (1.0)	0.355
UL tremor occurence	25/25 (100%)	13/23 (57%)	<0.001
TETRAS UL tremor	12.0 (5.0)	2.0 (5.5)	<0.001
TWSTRS	0 (0)	31.5 (19.8)	<0.001
TWSTRS IA	0 (0)	4.0 (1.0)	<0.001

*Note*: Results are shown as median (IQR) or as a ratio of person numbers (percentage).

Abbreviations: ADL, activities of daily living; DT, dystonic tremor; ET, essential tremor; PS, performance subscale; TETRAS, the essential tremor rating assessment scale; TWSTRS, the toronto western spasmodic torticollis rating scale; TWSTRS IA, torticollis severity scale, maximal excursion; UL, upper limbs.

**Figure 1:**
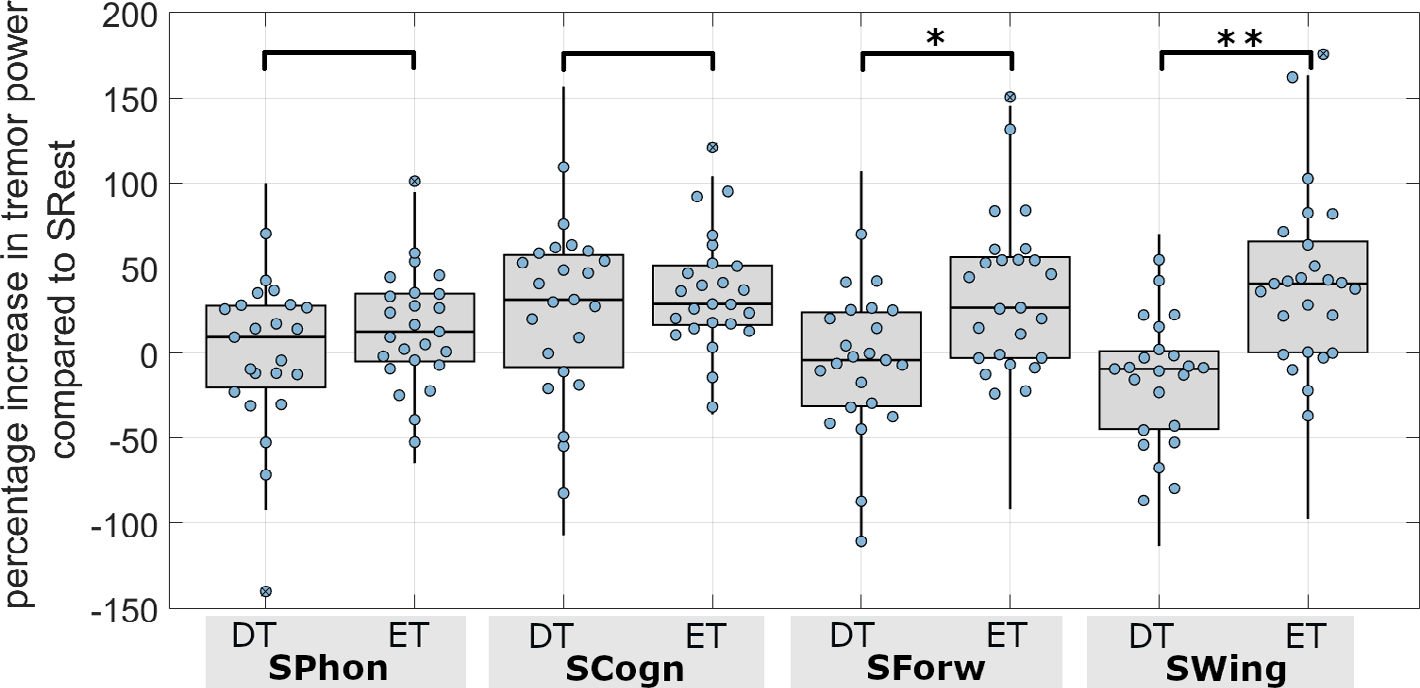
Percentage increase in head tremor power compared to head tremor power at rest. Crossed circles indicate outliers that were not included in the statistical analysis. Outliers were defined as values higher than Q3 + 1.5IQR or lower than Q1‐1.5IQR.* marks statistically significant results with corrected *P* < 0.0125, ** marks *P* < 0.001.

Regarding head tremor power at rest, there were no significant differences between ET and DT patients. Nonetheless, variations emerged concerning task‐related alterations in head tremor. During the cognitive task, head tremor was accentuated in both patients’ groups compared to the SRest condition. By contrast, the SWing and SForw tasks increased head tremor power solely in the ET group, with no significant changes observed in DT patients (Fig. 1). Similarly, a comparison of TETRAS PS head tremor scores in the SRest with SForw and SWing tasks demonstrated more frequent increases in ET patients than in DT patients (Table S1, Video 1).

**Video 1:** 83‐year‐old patient with ET; negative family history for tremor; first symptom was UL tremor at age 18; head tremor since age 60. In the SForw and SWing positions of the UL there is a visible increase in head tremor compared to the SRest position.

In the analysis of UL tremor, ET patients exhibited statistically significant differences in tremor power between the SRest and postural tremor‐inducing tasks (SWing and SForw), along with greater overall UL tremor power compared to DT patients. No variance in UL tremor power was observed when the hands were placed on the thighs (SRest, SPhon and SCogn tasks).

A strong coherence (above 0.7) was observed between head and UL tremor across all tasks in both patient groups. Coherence between the ULs was strong in most tasks (Table S2). No statistically significant differences in coherence were noted between ET and DT patients.

Head tremor frequency stability, assessed by TSI, did not significantly differ between the groups. Notably, statistical between‐group differences were solely observed in the frequency changes of UL tremor during the SWing and SForw tasks (Table S3).

## Discussion

Several clinical tests suggested for distinguishing between essential and dystonic head tremor^15^, ^17^ were found to be unreliable, particularly for common head tremors of mild to moderate intensity^16^, ^21^. Consequently, the identification of other easily applicable clinical tests would be beneficial for refining the clinical differential diagnosis and improving the targeted management of head tremor.

This study revealed differences between essential and dystonic head tremor in response to routine UL postural tremor tasks. During these tasks, head tremor power increased solely in ET patients, contrasting significantly with DT patients who showed no change. Conversely, no noticeable differences emerged during the cognitive task, where head tremor increased comparably in both groups. This observation could prove valuable in clinical scenarios where head tremor predominates but its etiology is uncertain.^16^, ^21^

From a pathophysiological perspective, the key question pertains to the mechanism driving the escalation of head tremor power during postural tasks. Is it primarily due to mechanical transfer of task‐activated UL tremor to the head, or does it involve activation of a common tremor generator? Alternatively, could head tremor amplification result from a more intricate mechanism involving sensory feedback? Although the close coherence between head and UL tremor might suggest a straightforward mechanical transmission of tremor, this alone may not fully explain the observed increase in head tremor amplitude. Central neurogenic tremor is known to be amplified when its frequency aligns with the oscillation frequency of the mechanical‐reflex system.^22^ Hence, the power of head tremor in ET patients would increase if the oscillations approached the resonance frequency of the system comprising the head, neck, and fixed upper limbs during postural task execution. However, further testing, including weight‐loading, would be needed to confirm this assumption.

Of note, besides action tremor, nearly half of the ET patients also had resting tremor, and three others showed mild head deviation. According to current classification criteria, the presence of such soft signs characterizes the ET‐plus category,^1^, ^23^ indicating an increased likelihood of alternative tremor syndromes.^24^ However, we observed UL position‐related changes in head tremor in both patients with and without additional soft signs. Further research could explore in more detail whether the presence of soft signs contributes to the effect of UL positioning.

Several limitations merit acknowledgement. Firstly, the study was conducted on a routine clinical sample primarily comprising cases with mild to moderate tremor severity. Validation in a larger population, including individuals with severe tremor, is warranted. Secondly, we did not investigate all head positions recommended for accelerometric assessment of head tremor.^25^ Instead, patients were instructed to maintain a relaxed head position without resisting tremor, which we deemed suitable for evaluating the effect of UL positions on head tremor. Thirdly, we cannot completely exclude the possibility that the difference found between DT and ET patients in response to UL position was influenced by previous BTX injections, which, with one exception, were received only by DT patients. However, it is unlikely that this played a role because the median interval since the last dose exceeded 4 months, commonly considered the time after which the effect of BTX injection wears off. In addition, outpatient records revealed similar head tremor subscores at both the initiation of treatment and before the study examination (see Supplementary Methods). Lastly, while statistically significant differences between ET and DT were observed, their clinical applicability for individual diagnosis may be limited due to overlapping parameter values between the groups.

In summary, inducing postural arm tremor can help differentiate the underlying causes of head tremor, potentially facilitating diagnosis and clinical assessment of patients with both essential and dystonic head tremor.

## Author Roles

(1) Research project: A. Conception, B. Organization, C. Execution; (2) Statistical Analysis: A. Design, B. Execution, C. Review and Critique; (3) Manuscript Preparation: A. Writing of the first draft, B. Review and Critique.

T.H.: 1B, 1C, 2B, 3A.

P.H.: 1B, 1C, 3B.

A.P.: 1C.

O.U.: 1C.

J.R.: 3B.

R.K.: 1A, 2A, 2C, 3B.

E.R.: 1A, 1B, 2C, 3B.

## Disclosures

**Ethical Compliance Statement:** An institutional ethics committee of General University Hospital in Prague approved the study. All patients declared their consent in written form. We confirm that we have read the Journal's position on issues involved in ethical publication and affirm that this work is consistent with those guidelines.

**Funding Sources and Conflict of Interest:** This work was supported by project National Institute for Neurological Research (Programme EXCELES, ID Project No. LX22NPO5107) and funded by the European Union—Next Generation EU and by the General University Hospital in Prague, MH CZ‐DRO‐VFN No. 64165. No other funding was received for this work. The authors declare that there are no conflicts of interest relevant to this work.

**Financial Disclosures for the previous 12 months:** The authors declare that there are no additional disclosures to report.

## Supporting information


**Data S1.** Supplementary Methods: Contains details on botulinum toxin treatment in cervical dystonia patients, as well as detailed information on tremor data analysis and statistical methods.

**Figure S1.** Positions of probands during measurement. Tasks from left to right: rest position (tasks SRest, SPhon and SCogn); task SWing; task SForw.

**TABLE S1.** Comparison of head tremor subscore ratings from video recordings (TETRAS PS item 1). SForw and SWing scores are compared to SRest score, the results are shown as a ratio of the number of persons (percentage).
**TABLE S2.** Coherence of tremor. The results are shown as median (IQR) for each task for ET and DT separately. Coherence was calculated between head and right arm (H:R), head and left arm (H:L) and between arms (R:L). The coherence values were all statistically significant at the significance level alpha 0.0017.
**TABLE S3.** Comparison of tremor stability index between ET and DT. The results are shown as median (IQR) and groupwise comparisons (*p*‐values), * marks statistically significant differences with alpha = 0.0125.

## Data Availability

The data that support the findings of this study are available from the corresponding author upon reasonable request.
