# Supplementary material for: Differentiating Essential and Dystonic Head Tremor: Exploring Arm Position Effects
Source: Mov Disord Clin Pract. 2024 Nov 15;12(1):71–5. doi: 10.1002/mdc3.14269 (PMC11736889; doi:10.1002/mdc3.14269)
Supplement: Supplementary file 1 — Data S1. Supplementary Methods: Contains details on botulinum toxin treatment in cervical dystonia patients, as well as detailed information on tremor data analysis and statistical methods. [file MDC3-12-71-s003.docx]

**Supplementary** **Methods**

Patients

According to the outpatient records, the median duration of BTX treatment in patients with cervical dystonia was 10 years (range 2 - 22 years), with a median of 27 BTX administrations (range 6 – 73). The median interval since the last BTX dose was 131 days (range 98 - 910 days). The median Tsui cervical dystonia scale score (1) was 12 (IQR 8.0) before the first BTX dose and 10 (IQR 6.0) at the last visit prior to the study. The median Tsui head tremor subscore was 2 (IQR 1.0) at the start of therapy and 2 (IQR 1.0) at the last visit.

Data analysis

Data were analyzed using custom-written software in the MatLab (The Mathworks, Massachusetts, USA). All data obtained from inertial measurement units were preprocessed including the removal of the gravitational component of the acceleration data. Dominant frequency and power of tremor were calculated. Dominant frequency values ranged between 2 – 12 Hz. The coherence of tremor was calculated as the dominant peak in the coherence spectrum between 2 and 12 Hz. The coherence spectrum was calculated from the first component of the PCA for extracting the tremor signal from the 3D accelerometer.

The head tremor ratings by the two raters were strongly correlated, differing by a maximum of 1 point for the SRest task (Kendall’s τ = 0.94, 95 % CI = [0.87; 0.99]), SForw (Kendall’s τ = 0.90, 95 % CI = [0.81; 0.97]) and the SWing task (Kendall’s τ = 0.68, 95 % CI = [0.56; 0.77]).

Statistics

For groupwise comparisons, a two-sample independent t-test was used; if the data were not normally distributed, the Wilcoxon rank-sum test was used. For groupwise comparisons, a two-sample independent t-test was used; if the data were not normally distributed, the Wilcoxon rank-sum test was applied. For pairwise comparisons, a paired t-test or Wilcoxon signed rank test was used, depending on the normality of the data. The overall significance level was set at 0.05. Fisher’s exact test was used for categorical variables. Outliers higher than Q3 + 1.5 IQR or lower than Q1 - 1.5 IQR were excluded from statistical evaluation. The Bonferroni correction was applied to control for the bias associated with multiple comparisons, adjusting the significance level to 0.0125 for the head tremor power comparison, 0.006 for clinical scores, and 0.0017 for signal coherence. All statistical analyses were performed in MatLab.

Reference

1. Tsui JKC, Eisen A, Stoessl AJ, Calne S, Calne DB. Double-blind study of botulinum toxin in spasmodic torticollis. Lancet 1986;2(8501):245-7.
