# Supplementary figures and images for: Differentiating Essential and Dystonic Head Tremor: Exploring Arm Position Effects
Source: Mov Disord Clin Pract. 2024 Nov 15;12(1):71–5. doi: 10.1002/mdc3.14269 (PMC11736889; doi:10.1002/mdc3.14269)

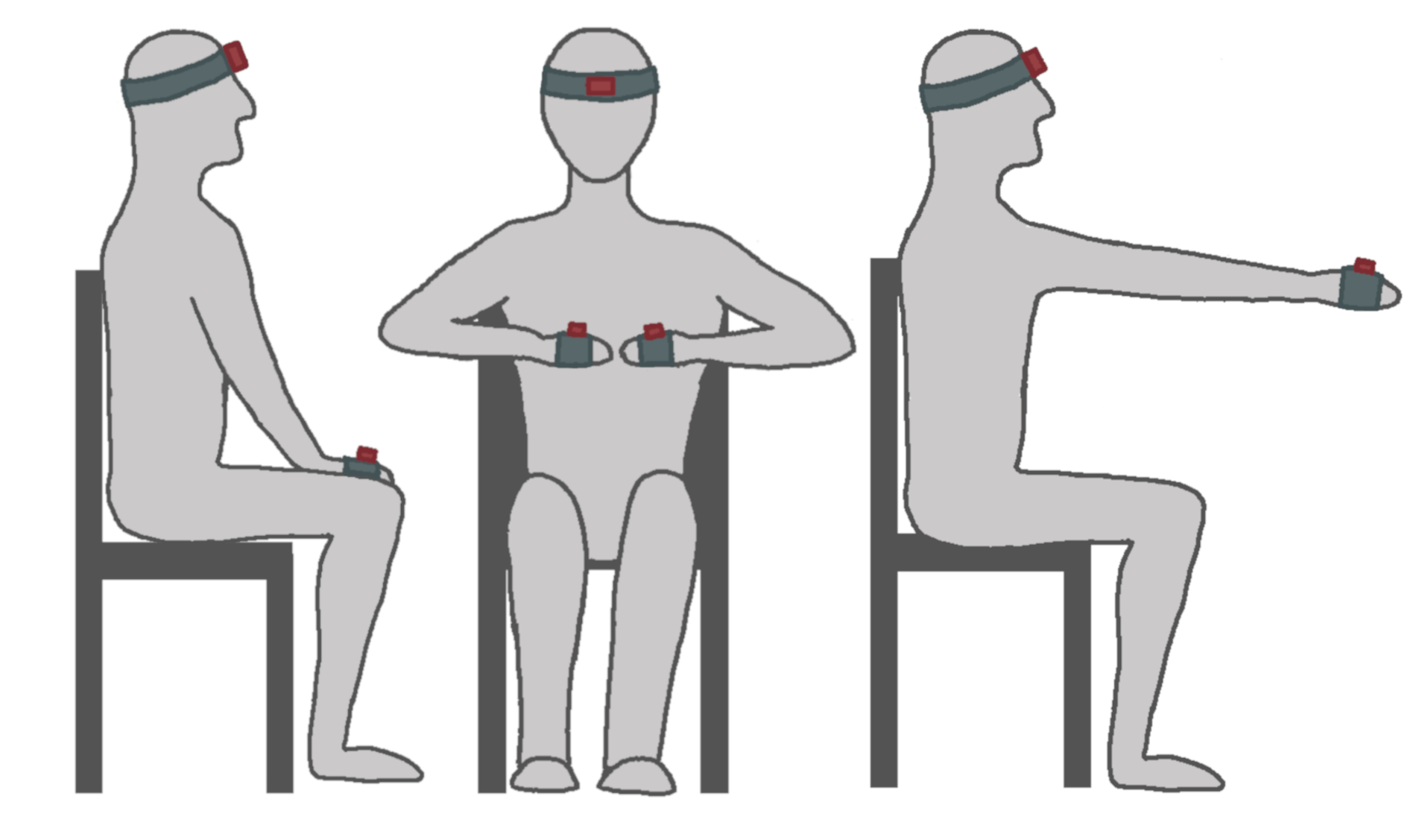

Supplement: Supplementary file 2 — Figure S1. Positions of probands during measurement. Tasks from left to right: rest position (tasks SRest, SPhon and SCogn); task SWing; task SForw. [file MDC3-12-71-s002.png]
