# Supplementary material for: Differentiating Essential and Dystonic Head Tremor: Exploring Arm Position Effects
Source: Mov Disord Clin Pract. 2024 Nov 15;12(1):71–5. doi: 10.1002/mdc3.14269 (PMC11736889; doi:10.1002/mdc3.14269)
Supplement: Supplementary file 3 — TABLE S1. Comparison of head tremor subscore ratings from video recordings (TETRAS PS item 1). SForw and SWing scores are compared to SRest score, the results are shown as a ratio of the number of persons (percentage). TABLE S2. Coherence of tremor. The results are shown as median (IQR) for each task for ET and DT separately. Coherence was calculated between head and right arm (H:R), head and left arm (H:L) and between arms (R:L). The coherence values were all statistically significant at the significance level alpha 0.0017. TABLE S3. Comparison of tremor stability index between ET and DT. The results are shown as median (IQR) and groupwise comparisons (p‐values), * marks statistically significant differences with alpha = 0.0125. [file MDC3-12-71-s001.docx]

**Supplementary Tables**

Suppl. Tab. 1. Comparison of head tremor subscore ratings from video recordings (TETRAS PS item 1).

|  | **SForw** | | **SWing** | |
| --- | --- | --- | --- | --- |
|  | **ET** | **DT** | **ET** | **DT** |
| **increase of head tremor** | 16/25 (64 %) | 4/23 (17 %) | 18/25 (72 %) | 2/23 (9 %) |
| **no change** | 8/25 (32 %) | 18/23 (78 %) | 4/25 (16 %) | 14/23 (61 %) |
| **decrease of head tremor** | 1/25 (4 %) | 1/23 (4 %) | 3/25 (12 %) | 7/23 (30 %) |

Note: SForw and SWing scores are compared to SRest score, the results are shown as a ratio of person numbers (percentage).

Suppl. Tab. 2. Coherence of tremor.

| **Group** | **Comparison** | **SRest** | **SPhon** | **SCogn** | **SForw** | **SWing** |
| --- | --- | --- | --- | --- | --- | --- |
| **ET** | **H:R** | 0.79 (0.16) | 0.80 (0.18) | 0.70(0.22) | 0.79 (0.24) | 0.86 (0.18) |
|  | **H:L** | 0.84 (0.15) | 0.72 (0.20) | 0.73 (0.17) | 0.72 (0.18) | 0.81 (0.17) |
|  | **R:L** | 0.90 (0.15) | 0.85 (0.19) | 0.75 (0.26) | 0.69 (0.26) | 0.74 (0.21) |
| **DT** | **H:R** | 0.92 (0.19) | 0.82 (0.19) | 0.83 (0.25) | 0.78 (0.25) | 0.73 (0.22) |
|  | **H:L** | 0.87 (0.19) | 0.87 (0.20) | 0.78 (0.24) | 0.80 (0.16) | 0.80 (0.26) |
|  | **R:L** | 0.91 (0.12) | 0.88 (0.20) | 0.83 (0.20) | 0.76 (0.21) | 0.67 (0.15) |

Note: The results are shown as median (IQR) for each task for ET and DT separately. Coherence was calculated between head and right arm (H:R), head and left arm (H:L) and between arms (R:L). The coherence values were all statistically significant at the significance level alpha 0.0017.

Suppl. Tab. 3 Comparison of tremor stability index between ET and DT.

|  |  | **SRest** | **SPhon** | **SCogn** | **SForw** | **SWing** |
| --- | --- | --- | --- | --- | --- | --- |
| **head** | **ET** | 1.39 (0.92) | 1.53 (0.81) | 1.68 (0.94) | 0.94 (0.94) | 0.83 (1.14) |
|  | **DT** | 0.95 (0.94) | 1.48 (1.16) | 1.19 (1.04) | 1.17 (1.10) | 1.45 (0.89) |
|  | p-values | 0.522 | 0.519 | 0.146 | 0.105 | 0.124 |
| **right hand** | **ET** | 1.43 (0.63) | 1.32 (0.86) | 1.34 (0.76) | 0.83 (1.03) | 0.56 (1.04) |
|  | **DT** | 1.52 (1.01) | 1.66 (1.15) | 1.61 (0.87) | 1.34 (0.67) | 1.62 (0.83) |
|  | p-values | 0.518 | 0.292 | 0.436 | 0.025 | **0.002*** |
| **left hand** | **ET** | 1.31 (0.87) | 1.40 (0.68) | 1.37 (0.83) | 1.10 (0.78) | 0.74 (0.63) |
|  | **DT** | 1.56 (1.06) | 1.82 (1.18) | 1.61 (0.79) | 1.42 (0.81) | 1.78 (0.83) |
|  | p-values | 0.724 | 0.406 | 0.540 | **0.007*** | **<0.001*** |

Note: The results are shown as median (IQR) and groupwise comparisons (p-values), * marks statistically significant differences with alpha=0.0125.
